# Polypeptide N-Acetylgalactosaminyltransferase 14 (GALNT14) as a Chemosensitivity-Related Biomarker for Osteosarcoma

**DOI:** 10.1155/2023/1083423

**Published:** 2023-02-02

**Authors:** Qiong Xi, Jinqi Ma

**Affiliations:** ^1^Department of Pediatrics, The Third Xiangya Hospital of Central South University, Changsha, China; ^2^Department of Blood Transfusion, The Third Xiangya Hospital of Central South University, Changsha, China

## Abstract

**Purpose:**

Osteosarcoma is the most common primary bone tumor. Polypeptide N-acetylgalactosaminyltransferase 14 (GALNT14), a member of the N-acetylgalactosaminyltransferase family, has been considered to be associated with various cancers. However, its role in osteosarcoma remains unknown. Here, we aimed to explore the expression and potential mechanism of GALNT14 in osteosarcoma through bioinformatics analysis and in vitro experiments.

**Methods:**

We investigated GALNT14 expression in osteosarcoma using GEO, the TIMER database, and clinical samples. Protein-protein interaction (PPI) network analysis on GALNT14 was performed by STRING. TARGET was used to identify differentially expressed genes (DEGs) between high and low GALNT14 expression. The correlation between GALNT14 and cuproptosis-related genes in osteosarcoma was analyzed by R language. The prognostic significance of GALNT14 was examined by Kaplan–Meier survival analysis. Additionally, we inhibited GALNT14 function in an osteosarcoma cell line by transfecting siRNA and subsequently explored the effect on drug sensitivity by CCK-8, clonogenic assay, and flow cytometry.

**Results:**

GALNT14 was significantly elevated in osteosarcoma tissue, osteosarcoma cell lines, and metastatic osteosarcoma. PPI analysis revealed that GALNT14 was associated with MUC7, MUC13, MUC5AC, C1GALT1, MUC15, MUC16, MUC1, MUC4, MUC21, and MUC17. In the high GALNT14 expression group, we discovered 81 upregulated DEGs and 73 downregulated DEGs. Functional enrichment analysis of DEGs showed significant enrichment in the Wnt, TGF-*β*, Hippo, PI3K signaling pathways and cell adhesion molecules. Expression of cuproptosis-related genes was closely related in osteosarcoma, and GALNT14 expression was significantly positively correlated with FDX1, a key regulator of cuproptosis. Kaplan–Meier survival showed that GALNT14 was linked to poor overall survival and disease-free survival in osteosarcoma. *In vitro* experiments suggested that GALNT14 was associated with chemotherapy resistance in osteosarcoma.

**Conclusion:**

We identified a GALNT family gene, GALNT14, that was highly expressed in osteosarcoma. This gene was closely associated with metastasis, progression, cuproptosis-related genes, and chemosensitivity of osteosarcoma, and showed correlation with poor overall survival and disease-free survival in osteosarcoma.

## 1. Introduction

Osteosarcoma, the most prevalent primary bone cancer, mostly occurs in children and adolescents, with the second highest incidence among older adults [1]. Multiagent chemotherapy combined with surgical resection is the standard treatment for patients with localized disease, and it results in long-term survival rates of approximately 70% [2]. Metastasis contributes to more than 90% of all cancer-related deaths, including osteosarcoma [3]. The lung is the site of more than 75% of osteosarcoma metastases, and most deaths from osteosarcoma are attributed to lung metastases. It is well understood how tumor cell dispersion and metastatic colonization of distant sites physically occur [4]. Whole-genome sequencing has elucidated many genes responsible for the metastatic progression of osteosarcoma. However, the underlying mechanisms are not well defined. Understanding the underlying mechanisms of the progression and metastasis of osteosarcoma may help to improve the prognosis.

The enzymes that make up the N-acetylgalactosaminyltransferase (GALNT) family add an N-acetylgalactosamine (GalNAc) to a threonine or serine residue of a mucin-type protein to start the process of O-glycosylation [5]. Thomsen-nouvelle (Tn) antigens, which are well-knowncancer-associated molecules, are synthesized largely as a result of this process [6]. There are 20 members in the GALNT family, ranging from GALNT1 to GALNT14 and from GANLTL1 to GANLTL6 [5]. The modification of O-glycosylation by GALNTs may impact a variety of biological functions associated with cancer, including tumor development, proliferation, and migration [7].

The GALNT14 gene, which is over 228 kb in length, is located on chromosome 2p23.1. The 552-amino acid type II membrane protein GALNT14 has a catalytic domain, a transmembrane domain, a stem region, and an N-terminal cytoplasmic domain [6]. GALNT14 has been found in various human tissues since it was first discovered in the gastric cancer cell line MKN45 in 2003 [8]. Further, the roles of GALNT14 in many malignancies have been identified, including the modification of migration characteristics, transformation of tissue invasiveness, and change in apoptotic signaling [9]. Clinically, GALNT14 has been proposed as a biomarker for anticancer therapy and prognosis [9]. However, the function of GALNT14 in osteosarcoma is still unclear.

In this study, we analyzed the expression of GALNT14 in osteosarcoma using expression data found in public databases. Then protein-protein interaction (PPI) analysis, GO/KEGG enrichment analysis, and correlation analysis of cuproptosis-related genes preliminarily revealed the potential function of GALNT14. Finally, we assessed the significance of GALNT14 in chemosensitivity and prognosis in osteosarcoma.

## 2. Methods

### 2.1. Differential Expression Analysis

The microarray transcriptome data were collected from three osteosarcoma datasets (GSE12865, GSE11414, and GSE21257) in the Gene Expression Omnibus (GEO, https://www.ncbi.nlm.nih.gov/geo) database. The three datasets contained samples from osteosarcoma tumor samples and normal human osteoblasts, osteosarcoma and human osteoblast cell lines, and osteosarcoma patients who developed metastases or not, respectively. GEO2R was used for GALNT14 expression analysis [10]. The Tumor Immune Estimation Resource (TIMER, https://cistrome.shinyapps.io/timer/) is a trustworthy and practical database that offers extensive gene expression data for a variety of cancer types. Using the DiffExp module of the TIMER database, we assessed the levels of GALNT14 in adjacent normal tissues and cancerous tissues across cancers [11].

### 2.2. Functional Enrichment Analysis

With online PPI data obtained from the STRING database (https://cn.string-db.org/) [12], we examined the GALNT14 PPI network.

Gene expression data of 98 osteosarcoma patients were obtained from the Therapeutically Applicable Research to Generate Effective Treatments (TARGET, https://ocg.cancer.gov/programs/target) database, an open database for childhood cancers. To examine the mRNA that differed in expression between groups (high GALNT14 expression vs. low GALNT14 expression), we used the limma package in the R programming language. The criterion for the differential expression of mRNAs was established as “adjusted *p* < 0.05 and Log2 (fold change) > 1 or Log2 (fold change) ≤1.”

The differential expression data were evaluated by functional enrichment in order to better support the underlying function of GALNT14. Gene Ontology (GO) is a popular approach for annotating genes with functions, particularly molecular function (MF), biological pathways (BP), and cellular components (CC). The Kyoto Encyclopedia of Genes and Genomes (KEGG) enrichment analysis is a useful tool for learning about gene functions and related high-level genome functional data. The ClusterProfiler package (version: 3.18.0) in R was used to examine the GO function and the enriched KEGG pathway of possible targets in order to better understand the carcinogenesis of GALNT14. The boxplot was created using the R package ggplot2. The heatmap was created using the R software's pheatmap package [13].

### 2.3. The Correlations among Cuproptosis-Related Genes and GALNT14

The cuproptosis-related genes (CDKN2A, FDX1, DLD, DLAT, LIAS, GLS, LIPT1, MTF1, PDHA1, and PDHB) [14] and GALNT14 expression data were obtained from the TARGET database. A parametric (Pearson) or nonparametric (Spearman) test method was employed for the correlation analysis based on the data normality test. Next, the correlations between cuproptosis-related genes and GALNT14 were displayed via the R software package heatmap. The R software package circlize (version 0.4.12) was used to display the chord diagram. The correlation between quantitative variables without a normal distribution was described using Spearman's correlation analysis. Statistics were considered significant for *p* values < 0.05.

### 2.4. Kaplan–Meier Survival Analysis of GALNT14

The gene expression and clinical information of 98 individuals with osteosarcoma were retrieved from the TARGET database. The difference in overall survival (OS) and disease-free survival (DFS) between the high GALNT14 expression group and the low GALNT14 expression group was compared using the log-rank test. *p* values and the hazard ratio (HR) with a 95% confidence interval (CI) were calculated for Kaplan–Meier curves using log-rank testing and univariate Cox proportional hazards regression. All analyses were performed in R version 4.0.3. Statistics were considered significant if *p* < 0.05.

### 2.5. Clinical Tissues and Cell Culture

Normal human osteoblasts and osteosarcoma tumor samples were obtained from healthy donors or patients with osteosarcoma at Third Xiangya Hospital. Fresh tissues were preserved with liquid nitrogen. All patients provided informed consent. Human osteosarcoma cell lines MG-63 and U-2 osteosarcoma were purchased from the American Type Culture Collection (ATCC; Manassas, VA, United States). Cell culture was performed based on the recommended protocols. Osteosarcoma cell lines were transfected with siRNA (5′-CCA UCC AGA AGG GCA AUA UTT-3′ (sense) and 5′- AUA UUG CCC UUC UGG AUG GTT-3′ (antisense)) using LipofectamineTM 3000 (Invitrogen, MA, United States) [15].

### 2.6. Cell Viability Assay and Clonogenic Assay

The cell counting kit-8 (CCK-8) assay was used to determine the viability of the cells. 96-well plates were seeded with MG-63 or U-2 osteosarcoma cells. Following adhesion, the cells were given the indicated concentrations and times of cisplatin or doxorubicin. Each well was then filled with 10 *μ*l of CCK-8 reagent, and each was cultured at 37°C for 1 hour. The optical density of the cell lysates at 450 nm was measured in order to calculate the relative number of surviving cells.

For colony formation assays, MG-63 or U-2 osteosarcoma cells were seeded at 500 cells/well in 6-well plates and treated with various concentrations of cisplatin or doxorubicin for 14 days. After that, cells were fixed with 4% paraformaldehyde, followed by three washes in PBS and two washes in ddH_2_O.

### 2.7. Extraction of RNA and qPCR

Using the usual TRIzol (Invitrogen, United States) RNA extraction technique, total RNA was isolated from the cells. Using a DNA/RNA GeneQuant Calculator, the quantity of RNA samples was determined by UV absorbance at 260–280 nm (Amersham Biosciences, Piscataway, NJ, USA). Using the PrimeScript RT Reagent Kit, reverse transcription was carried out (Takara, China). Real-time qPCR was carried out with the use of the Brilliant II SYBR Green RT-qPCR kit. For RT-PCR, the following primers were utilized: GALNT14 (474 bp): sense 5′-ACCTGGACACCT TCACCTACAT-3′, antisense 5′- CCAATCTGCTCTCAACATTCC-3′; GAPDH (230 bp): sense 5′- CTCTCTGCTCCTCCTGTTCGACAG-3′, antisense 5′- GTGGAATCATATTGGAACATGT -3′.

### 2.8. Flow Cytometry

Apoptosis was measured using Annexin V/PI double-staining (KeyGEN BioTECH, China). Briefly, cells were incubated with the required conditions and then washed with PBS and trypsinized to get a single cell suspension. Next, 10^5^ cells/ml of suspension was suspended in 100 *μ*L binding buffer and stained with Annexin V/PI in the dark for 30 min. The analysis was performed with FACSVerse (BD Biosciences) and FlowJo software (Tree Star, United States).

### 2.9. Statistical Analyses

Each result was independently verified by at least three separate experiments, and all data were displayed as mean ± standard deviation (SD). To determine whether differences were statistically significant, two-sided Student's *t*-tests or analysis of variance tests were utilized. Statistics were considered to be significant at *p* < 0.05.

## 3. Results

### 3.1. GALNT14 Expression in Osteosarcoma and Pan-Cancer

**Figure 1:**
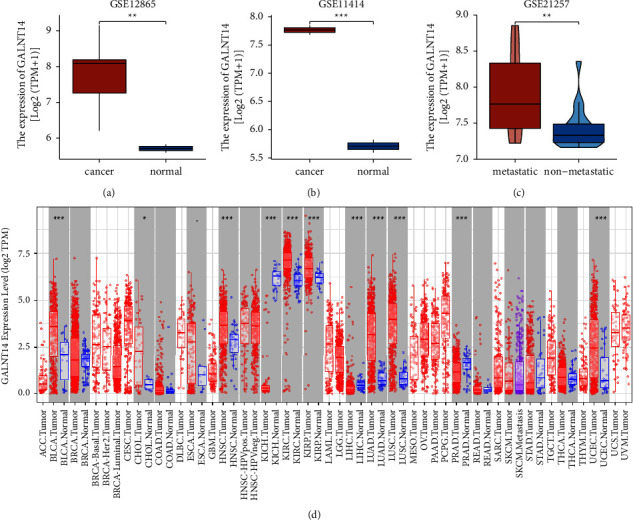
GALNT14 expression in osteosarcoma and pan-cancer. (a) GALNT14 expression data in an osteosarcoma from GSE12865. (b) GALNT14 expression data in osteosarcoma cell line from GSE11414. (c) GALNT14 expression data in metastatic osteosarcoma from GSE21257. (d) GALNT14 pan-cancer expression in TIMER. ^*∗∗*^*p* < 0.01, ^*∗∗∗*^*p* < 0.001.

GSE12865 provided the expression profiles of osteosarcoma tumor samples and normal human osteoblasts. As shown in Figure 1(a), GALNT14 expression was significantly elevated in osteosarcoma (*p*=0.002). We also observed upregulation of GALNT14 in the osteosarcoma cell line U2OS compared to GALNT14 expression in the human osteoblast cell line HOB (*p* < 0.001) (Figure 1(b)). Furthermore, osteosarcoma at the metastatic stage showed an increased GALNT14 level compared to that at the nonmetastatic stage (*p*=0.004) (Figure 1(c)). The levels of GALNT14 across cancers were examined using the TIMER database. Compared with adjacent normal tissues, GALNT14 presented with a significantly higher expression in the tumor tissues of bladder urothelial carcinoma, cholangiocarcinoma, head and neck squamous cell carcinoma, kidney renal clear cell carcinoma, kidney renal papillary cell carcinoma, lung adenocarcinoma, lung squamous cell carcinoma, and uterine corpus endometrial carcinoma. However, kidney chromophobe, liver hepatocellular carcinoma, and prostate adenocarcinoma had lower GALNT14 expression than adjacent normal tissues (Figure 1(d)). All the *p* values of pan-cancer analysis were summarized in Supplementary Table 1.

### 3.2. PPI and Differentially Expressed Genes Associated with GALNT14

**Figure 2:**
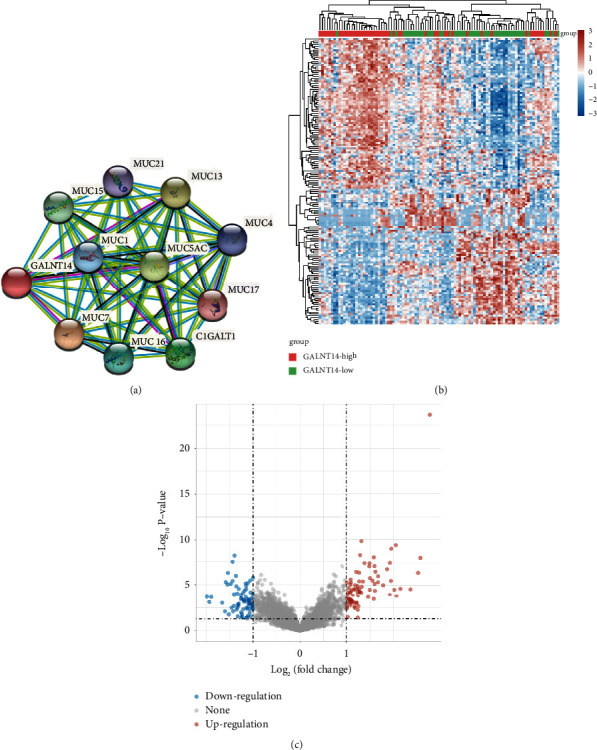
GALNT14 PPI network and coexpression genes in osteosarcoma. (a) PPI network of GALNT14. Differentially expressed genes associated with GALNT14 level showing in heatmap (b) and volcano plot (c).

**Figure 3:**
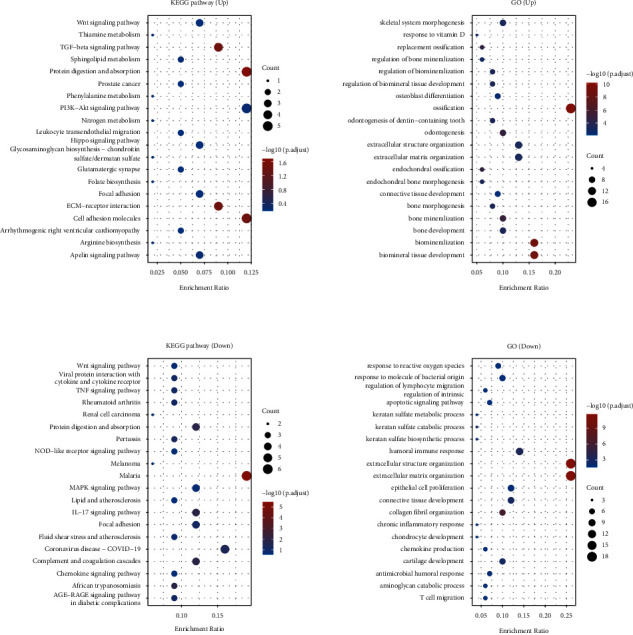
GO and KEGG enrichment analysis of genes associated with GALNT14 levels in osteosarcoma.

We used the STRING database to analyze the GALNT14 PPI network and revealed a link between GALNT14 and MUC7, MUC13, MUC5AC, C1GALT1, MUC15, MUC16, MUC1, MUC4, MUC21, and MUC17 (Figure 2(a)). We analyzed differentially expressed genes associated with GALNT14 levels using the osteosarcoma expression profile from the TARGET database, which contains the largest sample size of osteosarcoma. Differentially expressed genes were represented by a heatmap and a volcano plot (Figures 2(b) and 2(c)). In the high GALNT14 expression group, we discovered 81 upregulated genes and 73 downregulated genes. The functional enrichment analysis showed significant enrichment in pathways related to tumorigenesis and progression, such as the Wnt, TGF-*β*, Hippo, and PI3K signaling pathways. In addition, we also observed the enrichment of cell adhesion molecules, which may be closely related to tumor metastasis (Figure 3).

### 3.3. GALNT14 Was Associated with Cuproptosis-Related Genes in Osteosarcoma

**Figure 4:**
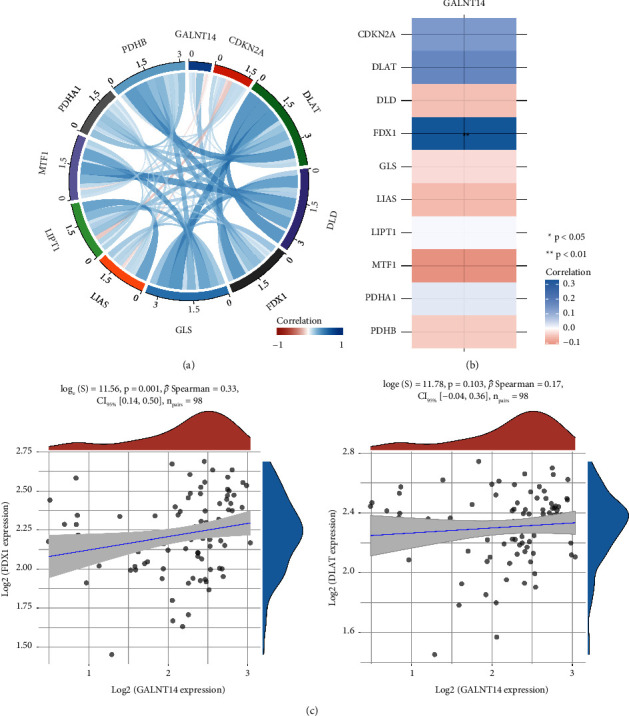
Correlation between GALNT14 and cuproptosis-related genes in osteosarcoma. (a) The correlation of expression of GALNT14 and 10 cuproptosis-related genes. (b) The correlation between GALNT14 and cuproptosis-related genes in osteosarcoma. (c) The correlation between GALNT14, FDX1, and DLAT in osteosarcoma.

We analyzed the correlation of the expression of GALNT14 and 10 cuproptosis-related genes by using the TARGET osteosarcoma dataset. As shown in Figure 4(a) and Supplementary Table 2, the expression levels of genes associated with cuproptosis were strongly correlated with osteosarcoma. Then the relationship between GALNT14 and cuproptosis-related genes was displayed separately. GALNT14 expression was significantly positively correlated with FDX1 (*R* = 0.33, *p* < 0.001), a key regulator of copper ionophore-induced cell death (Figure 4(b)). Additionally, there was some association between DLAT and GALNT14 expression (*R* = 0.17, *p*=0.103). The gene expression distributions are displayed in a dot plot (Figure 4(c))..

### 3.4. GALNT14 Was Associated with Poor OS and DFS in Osteosarcoma

**Figure 5:**
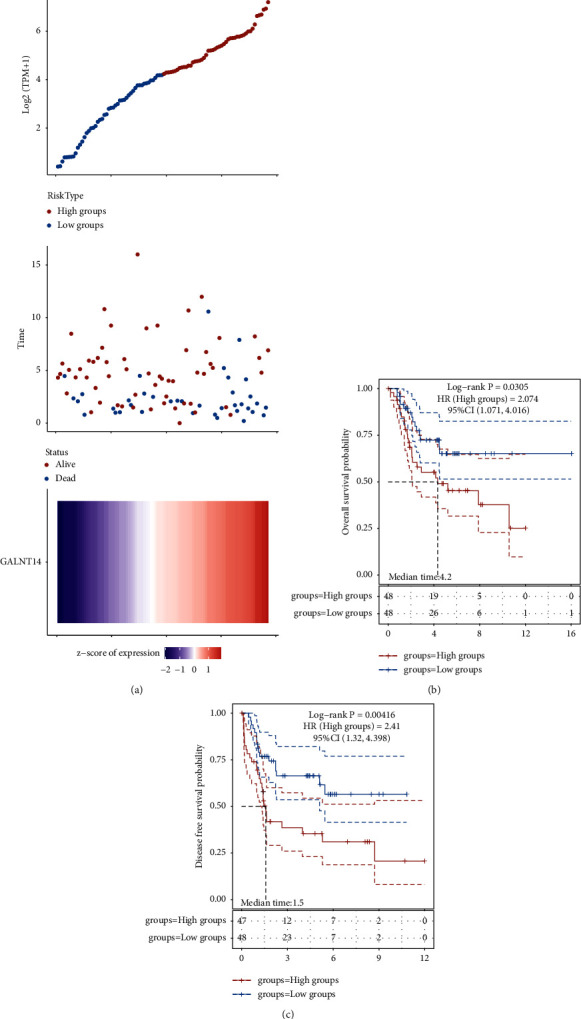
The OS significance of GALNT14 in osteosarcoma. (a) The gene expression, survival time, and OS survival status of the TARGET dataset. The top scatterplot represents gene expression from low to high. The scatter plot distribution represents the gene expression of different samples corresponding to the survival time and survival status. The bottom figure is the gene expression heatmap. (b) OS Kaplan–Meier survival analysis. (c) DFS Kaplan–Meier survival analysis. OS, overall survival; DFS, disease-free survival.

We first performed a detailed analysis of the impact of GALNT14 on OS in osteosarcoma. The correlation between gene expression and survival time and status is depicted in Figure 5(a); high GALNT14 expression was associated with poor OS in osteosarcoma. Kaplan–Meier survival curves also demonstrated this trend (*p*=0.0305, HR = 2.074, 95% CI (1.071, 4.016)) (Figure 5(b)). In addition, GALNT14 was also associated with poor DFS in osteosarcoma (*p*=0.00416, HR = 2.41, 95% CI (1.32, 4.398)) (Supplementary Figure 1 and Figure 5(c)).

### 3.5. GALNT14 Was Related to Chemosensitivity in Osteosarcoma

**Figure 6:**
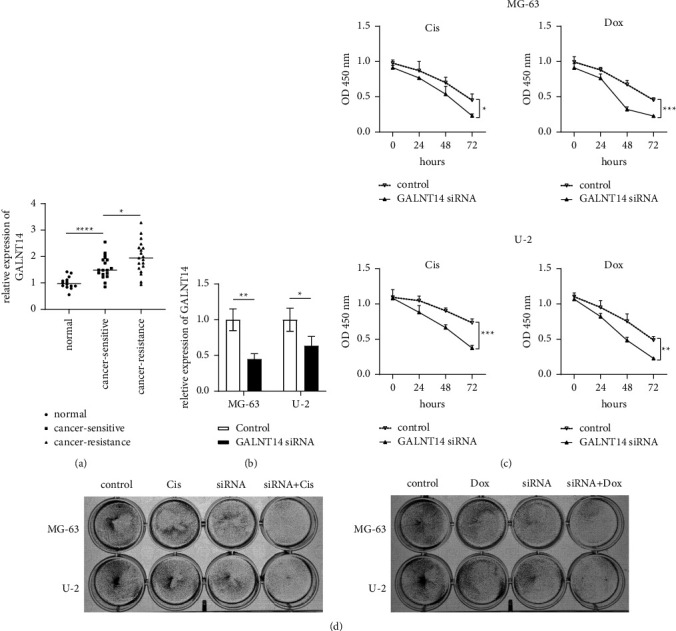
Interfering with GALNT14 expression promoted the inhibitory effect of cisplatin and doxorubicin on the proliferation of osteosarcoma cell lines. (a) The expression of GALNT14 in normal human osteoblasts, drug-sensitive osteosarcoma, and drug-resistant osteosarcoma. (b) GALNT14 siRNA transfection in osteosarcoma cell lines MG-63 and U-2. (c) MG-63 and U-2 OS cells were treated with Cis (20 *μ*mol/L) or Dox (0.2 *μ*g/mL) for 24 h. Then, cell viability was determined at 24 h, 48 h, and 72 h by the CCK-8 assay. (d) MG-63 or U-2 OS cells were seeded in six-well plates and treated with Cis (5 *μ*mol/L) and Dox (0.05 *μ*g/mL). The plates were imaged at 14 days to assess clones. Cis, cisplatin; Dox, doxorubicin. ^*∗*^*p* < 0.05, ^*∗∗*^*p* < 0.01, ^*∗∗∗*^*p* < 0.001, ^*∗∗∗∗*^*p* < 0.0001.

**Figure 7:**
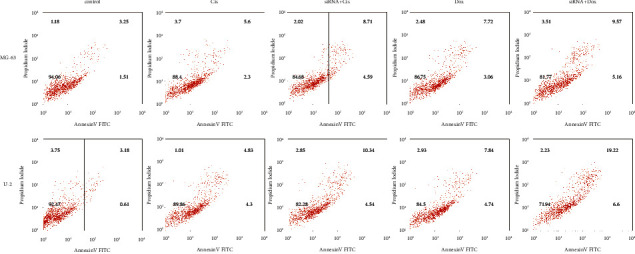
Interfering with GALNT14 expression promotes the apoptotic effect of cisplatin and doxorubicin on osteosarcoma cell lines. MG-63 and U-2 OS cells were treated with Cis (20 *μ*mol/L) or Dox (0.2 *μ*g/mL) for 24 h. Then, apoptosis was determined by flow cytometry. Cis, cisplatin; Dox, doxorubicin.

First, we measured the expression of GALNT14 in normal human osteoblasts, drug-sensitive osteosarcoma, and drug-resistant osteosarcoma by RT-qPCR. As shown in Figure 6(a), the expression of GALNT14 was increased in osteosarcoma compared with normal osteoblasts, especially in drug-resistant osteosarcoma. Then, we explored the effect of GALNT14 on drug sensitivity in vitro. A siRNA was used to inhibit the expression of GALNT14 in the osteosarcoma cell lines MG-6 and U-2 (Figure 6(b)). We found that inhibition of GALNT14 improved sensitivity to the chemotherapeutics cisplatin and doxorubicin in MG-6 and U-2 cell lines by detecting cell proliferation with CCK-8 (Figure 6(c)), and consistent trends were also observed in the clonogenic assay (Figure 6(d)). In addition, as shown in Figure 7, flow cytometry revealed that inhibition of GALNT14 resulted in an increased rate of apoptosis after cisplatin or doxorubicin treatment.

## 4. Discussion

The association between GALNT14 expression and cancer characteristics has been studied in multiple tumors. For example, GALNT14 was found to be overexpressed in most breast cancer tissues and associated with lung metastasis [16, 17]. Overexpression of GALNT14 was also found in ovarian cancer [15, 18]. In summary, previous reports have suggested abnormal expression of GALNT14 in cancer and metastatic tissues. Our study revealed for the first time the expression of GALNT14 in osteosarcoma. Consistently, increased expression of GALNT14 was identified in osteosarcoma. GALNT14 expression was also elevated in metastatic osteosarcoma compared to the nonmetastatic stage. Our results support the potential application of GALNT14 as a predictive biomarker for the diagnosis and metastasis of osteosarcoma.

Previous studies have suggested the significance of GALNT14 for the metastasis of a wide variety of cancers [19–21]. In this study, in addition to aberrant expression in metastatic osteosarcoma, GALNT14 was also found to interact with mucins (except for C1GALT1) by PPI analysis. Altered mucin glycosylation patterns during malignant transformation have been considered to promote cancer cell differentiation, proliferation, invasion, and metastasis [22]. Additionally, we observed that multiple cancer-related pathways were enriched through functional enrichment analysis of genes associated with GALNT14 levels, such as the Wnt, TGF-*β*, Hippo, and PI3K signaling pathways. The Wnt signaling pathway is one of the key cascades regulating development and has also been tightly associated with osteosarcoma [23]. TGF-*β*s have been considered to exhibit protumoral and promigratory effects on osteosarcoma [24]. The Hippo signaling pathway shows a close relationship with osteosarcoma and is a potential therapeutic target in the future [25]. These results reveal the underlying mechanism by which GALNT14 promotes tumorigenesis and metastasis in osteosarcoma.

The elimination of superfluous and damaged cells is achieved by the vital process of cell death. Apoptosis, necroptosis, and ferroptosis are a few examples of programmed and nonprogrammed cell death that have been uncovered in previous research [26]. Recently, abnormal copper ion elevations have been linked to a previously unidentified death pathway called cuproptosis [27]. Therefore, significant interest has developed in the association of cuproptosis-related genes with osteosarcoma. Our results showed that the expression of multiple cuproptosis-related genes was closely correlated with osteosarcoma. In addition, GALNT14 is also closely associated with the expression of FDX1, a key gene in cuproptosis. In summary, our results suggest a possible novel mechanism of GALNT14 involvement in osteosarcoma cell proliferation and death.

Prognostic significance is currently a hot topic in oncology research. Multiple genes have been determined to be associated with the prognosis of osteosarcoma, and some prognostic models have been established [28, 29]. However, the prognostic significance of GALNT14 in osteosarcoma remains unknown. Previous studies have reported an association between GALNT14 and the prognosis of a variety of cancers, including gastric cancer, cholangiocarcinoma, ovarian cancer, and breast cancer [18, 21, 30, 31]. Our research revealed a strong correlation between high GALNT14 expression and poor OS and DFS in osteosarcoma. This finding indicates the potential of GALNT14 as a prognostic biomarker for osteosarcoma.

Through an extensive repertoire of resistance mechanisms, tumors elude targeted cancer therapy. This is also one of the main reasons for the poor prognosis of tumors. Osteosarcoma is a kind of disease that is particularly resistant to chemotherapy. This disease, according to clinical trials, only responds to high doses of chemotherapy and rapidly acquires resistance [32, 33]. Chemotherapy resistance in osteosarcoma potentially correlates to drug build-up in the cell, DNA damage repair, intracellular detoxification, signal transduction, apoptosis, the tumor microenvironment, and immunity [34]. GALNT14 serves as an emerging marker for predicting therapeutic outcomes in multiple tumors [9], and it has also been identified to be related to paclitaxel resistance in lung adenocarcinoma [35]. Here, we reveal for the first time the significance of GALNT14 in the chemosensitivity of osteosarcoma.

There are some limitations to this study. For example, the molecular interaction of GALNT14 and the mechanism of GALNT14 on drug sensitivity need further experimental verification. In conclusion, we identified a GALNT family gene, GALNT14, that was highly expressed in osteosarcoma. GALNT14 was closely associated with metastasis, progression, cuproptosis-related genes, and the chemosensitivity of osteosarcoma. Finally, the poor prognostic significance of GALNT14 in osteosarcoma was also elucidated. This discovery is anticipated to offer fresh perspectives on osteosarcoma diagnosis and therapy.

## Data Availability

The data used to support the findings of this study are included within the article.

## Supplementary Materials

Supplementary Materials Supplementary Figure 1: the gene expression, survival time, and DFS survival status of the TARGET dataset. The top scatterplot represents gene expression from low to high. The scatter plot distribution represents the gene expression of different samples corresponding to the survival time and survival status. The bottom figure is the gene expression heatmap. Supplementary Table 1: *p* values of GALNT14 expression in pan-cancer analysis. Supplementary Table 2: the expression levels of cuproptosis-related genes were strongly correlated in osteosarcoma.
