# Supplementary material for: Polypeptide N-Acetylgalactosaminyltransferase 14 (GALNT14) as a Chemosensitivity-Related Biomarker for Osteosarcoma
Source: J Oncol. 2023 Feb 2;2023:1083423. doi: 10.1155/2023/1083423 (PMC10681776; doi:10.1155/2023/1083423)
Supplement: Supplementary Materials — Supplementary Figure 1: the gene expression, survival time, and DFS survival status of the TARGET dataset. The top scatterplot represents gene expression from low to high. The scatter plot distribution represents the gene expression of different samples corresponding to the survival time and survival status. The bottom figure is the gene expression heatmap. Supplementary Table 1: p values of GALNT14 expression in pan-cancer analysis. Supplementary Table 2: the expression levels of cuproptosis-related genes were strongly correlated in osteosarcoma. [file 1083423.f1.docx]

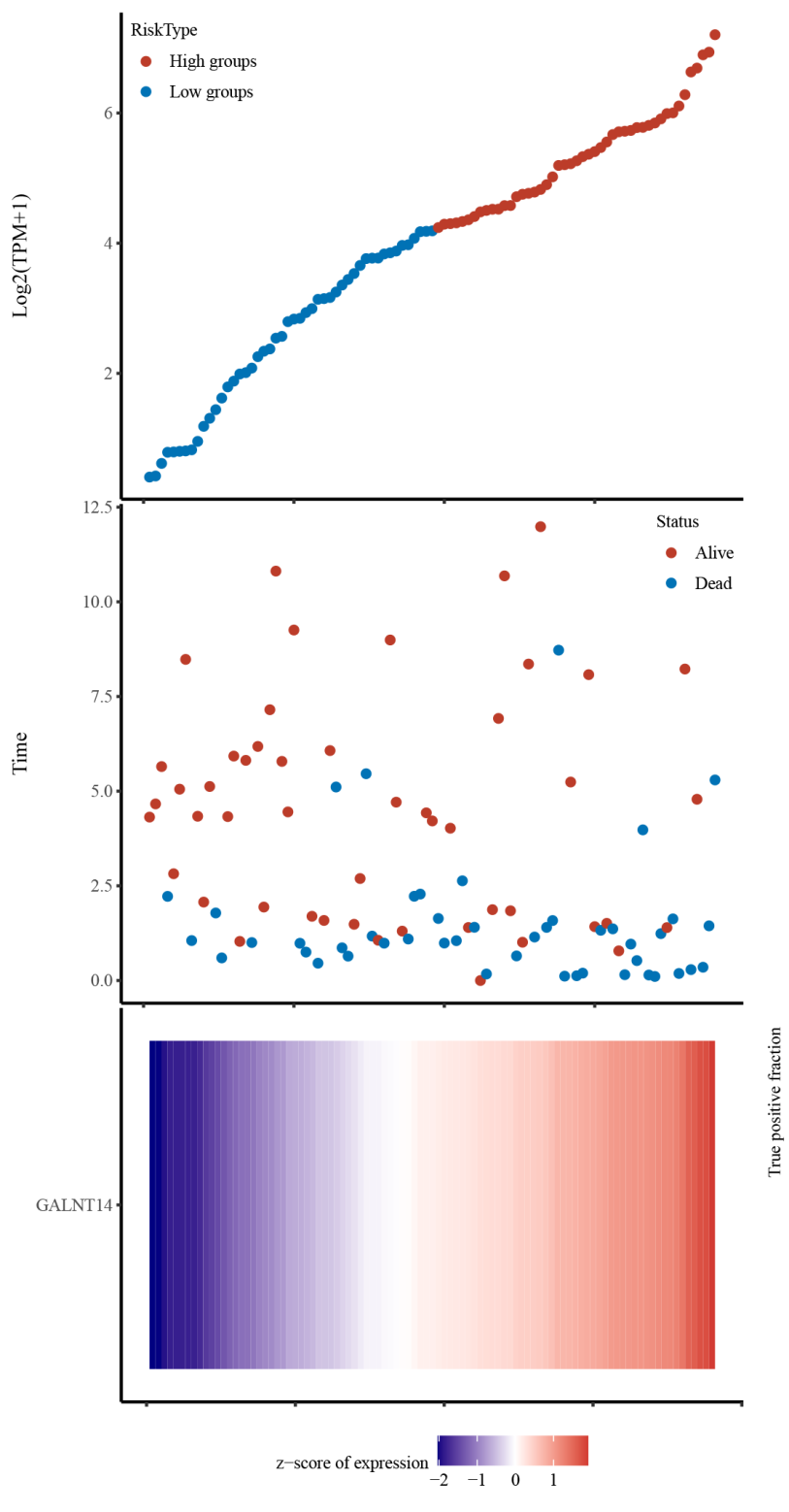


Supplementary Figure 1. The gene expression, survival time and DFS survival status of TARGET dataset. The top scatterplot represents the gene expression from low to high. The scatter plot distribution represents the gene expression of different samples corresponding to the survival time and survival status. The bottom figure is the gene expression heatmap.

Supplementary Table 1. P values of GALNT14 expression in pan-cancer analysis.

| Tumor | Nor | p |
| --- | --- | --- |
| BLCA.Tumor | BLCA.Normal | 0.000163 |
| BRCA.Tumor | BRCA.Normal | 0.473973 |
| CHOL.Tumor | CHOL.Normal | 0.014596 |
| COAD.Tumor | COAD.Normal | 0.124446 |
| ESCA.Tumor | ESCA.Normal | 0.075202 |
| HNSC-HPVpos.Tumor | HNSC-HPVneg.Tumor | 0.277904 |
| HNSC.Tumor | HNSC.Normal | 6.32E-06 |
| KICH.Tumor | KICH.Normal | 2.98E-13 |
| KIRC.Tumor | KIRC.Normal | 1.69E-23 |
| KIRP.Tumor | KIRP.Normal | 1.10E-05 |
| LIHC.Tumor | LIHC.Normal | 1.87E-07 |
| LUAD.Tumor | LUAD.Normal | 1.37E-22 |
| LUSC.Tumor | LUSC.Normal | 1.48E-23 |
| PRAD.Tumor | PRAD.Normal | 0.000367 |
| READ.Tumor | READ.Normal | 0.966865 |
| SKCM.Tumor | SKCM.Metastasis | 0.663351 |
| STAD.Tumor | STAD.Normal | 0.626565 |
| THCA.Tumor | THCA.Normal | 0.15592 |
| UCEC.Tumor | UCEC.Normal | 2.39E-05 |

Supplementary Table 2. The expression levels of genes associated to cuproptosis were strongly correlated in osteosarcoma.

| gene1 | gene2 | cor_spearman | p_spearman |
| --- | --- | --- | --- |
| GALNT14 | CDKN2A | 0.131266377 | 0.197613488 |
| GALNT14 | DLAT | 0.165668892 | 0.10304182 |
| CDKN2A | DLAT | 0.211917194 | 0.597207 |
| GALNT14 | DLD | -0.05403286 | 0.000901235 |
| CDKN2A | DLD | 0.074817181 | 0.781108926 |
| DLAT | DLD | 0.626213747 | 0.562245163 |
| GALNT14 | FDX1 | 0.330120052 | 0.948384512 |
| CDKN2A | FDX1 | 0.153019783 | 0.301030381 |
| DLAT | FDX1 | 0.610580877 | 0.826335683 |
| DLD | FDX1 | 0.256775625 | 0.679365289 |
| GALNT14 | GLS | -0.028428616 | 0.197613488 |
| CDKN2A | GLS | 0.189475228 | 0.03618777 |
| DLAT | GLS | 0.635318045 | 0.464060925 |
| DLD | GLS | 0.560137457 | 0.132515329 |
| FDX1 | GLS | 0.324841089 | 0.061677467 |
| GALNT14 | LIAS | -0.059248067 | 0.226262743 |
| CDKN2A | LIAS | -0.123347933 | 0.043802941 |
| DLAT | LIAS | 0.215015716 | 0.042856295 |
| DLD | LIAS | 0.239510612 | 0.065590934 |
| FDX1 | LIAS | 0.109232446 | 0.052940759 |
| GLS | LIAS | 0.294531683 | 0.10304182 |
| GALNT14 | LIPT1 | 0.006624205 | 0.03618777 |
| CDKN2A | LIPT1 | -0.20411351 | 5.35527E-12 |
| DLAT | LIPT1 | 0.335284254 | 2.46578E-11 |
| DLD | LIPT1 | 0.257336674 | 2.11431E-12 |
| FDX1 | LIPT1 | 0.167377541 | 0.033488953 |
| GLS | LIPT1 | 0.400263948 | 0.000738694 |
| LIAS | LIPT1 | 0.549656039 | 1.44568E-05 |
| GALNT14 | MTF1 | -0.105534622 | 0.002768057 |
| CDKN2A | MTF1 | 0.20501884 | 2.67719E-11 |
| DLAT | MTF1 | 0.422782421 | 0.597207 |
| DLD | MTF1 | 0.590230094 | 0.464060925 |
| FDX1 | MTF1 | 0.103201168 | 5.35527E-12 |
| GLS | MTF1 | 0.533972164 | 0.010702032 |
| LIAS | MTF1 | 0.165847407 | 2.01001E-09 |
| LIPT1 | MTF1 | 0.241614546 | 0.01753492 |
| GALNT14 | PDHA1 | 0.022448342 | 0.010526017 |
| CDKN2A | PDHA1 | 0.186746489 | 1.59485E-10 |
| DLAT | PDHA1 | 0.299160339 | 0.000536225 |
| DLD | PDHA1 | 0.343419467 | 1.18265E-08 |
| FDX1 | PDHA1 | 0.277508942 | 0.000901235 |
| GLS | PDHA1 | 0.151629912 | 0.132515329 |
| LIAS | PDHA1 | 0.19486895 | 2.46578E-11 |
| LIPT1 | PDHA1 | 0.098266486 | 0.010702032 |
| MTF1 | PDHA1 | 0.169086191 | 0.001100521 |
| GALNT14 | PDHB | -0.042276329 | 0.284313641 |
| CDKN2A | PDHB | 0.19611856 | 0.099488862 |
| DLAT | PDHB | 0.609713801 | 0.311904278 |
| DLD | PDHB | 0.537147193 | 0.005666009 |
| FDX1 | PDHB | 0.387563835 | 8.05648E-05 |
| GLS | PDHB | 0.416508872 | 0.781108926 |
| LIAS | PDHB | 0.218343757 | 0.061677467 |
| LIPT1 | PDHB | 0.240874982 | 2.11431E-12 |
| MTF1 | PDHB | 0.205847662 | 2.01001E-09 |
| PDHA1 | PDHB | 0.348545416 | 0.001100521 |
| GALNT14 | CDKN2A | 0.131266377 | 0.003241035 |
| GALNT14 | DLAT | 0.165668892 | 4.43322E-05 |
| CDKN2A | DLAT | 0.211917194 | 1.49511E-08 |
| GALNT14 | DLD | -0.05403286 | 0.136110581 |
| CDKN2A | DLD | 0.074817181 | 1.99146E-05 |
| DLAT | DLD | 0.626213747 | 0.562245163 |
| GALNT14 | FDX1 | 0.330120052 | 0.226262743 |
| CDKN2A | FDX1 | 0.153019783 | 0.033488953 |
| DLAT | FDX1 | 0.610580877 | 0.01753492 |
| DLD | FDX1 | 0.256775625 | 0.284313641 |
| GALNT14 | GLS | -0.028428616 | 0.003241035 |
| CDKN2A | GLS | 0.189475228 | 4.5846E-09 |
| DLAT | GLS | 0.635318045 | 0.102666033 |
| DLD | GLS | 0.560137457 | 0.054501507 |
| FDX1 | GLS | 0.324841089 | 0.030781018 |
| GALNT14 | LIAS | -0.059248067 | 0.948384512 |
| CDKN2A | LIAS | -0.123347933 | 0.043802941 |
| DLAT | LIAS | 0.215015716 | 0.000738694 |
| DLD | LIAS | 0.239510612 | 0.010526017 |
| FDX1 | LIAS | 0.109232446 | 0.099488862 |
| GLS | LIAS | 0.294531683 | 4.43322E-05 |
| GALNT14 | LIPT1 | 0.006624205 | 4.5846E-09 |
| CDKN2A | LIPT1 | -0.20411351 | 0.016539021 |
| DLAT | LIPT1 | 0.335284254 | 0.335728479 |
| DLD | LIPT1 | 0.257336674 | 0.016883374 |
| FDX1 | LIPT1 | 0.167377541 | 0.301030381 |
| GLS | LIPT1 | 0.400263948 | 0.042856295 |
| LIAS | LIPT1 | 0.549656039 | 1.44568E-05 |
| GALNT14 | MTF1 | -0.105534622 | 1.59485E-10 |
| CDKN2A | MTF1 | 0.20501884 | 0.311904278 |
| DLAT | MTF1 | 0.422782421 | 1.49511E-08 |
| DLD | MTF1 | 0.590230094 | 0.102666033 |
| FDX1 | MTF1 | 0.103201168 | 0.016539021 |
| GLS | MTF1 | 0.533972164 | 0.096032823 |
| LIAS | MTF1 | 0.165847407 | 0.042004633 |
| LIPT1 | MTF1 | 0.241614546 | 0.826335683 |
| GALNT14 | PDHA1 | 0.022448342 | 0.065590934 |
| CDKN2A | PDHA1 | 0.186746489 | 0.002768057 |
| DLAT | PDHA1 | 0.299160339 | 0.000536225 |
| DLD | PDHA1 | 0.343419467 | 0.005666009 |
| FDX1 | PDHA1 | 0.277508942 | 0.136110581 |
| GLS | PDHA1 | 0.151629912 | 0.054501507 |
| LIAS | PDHA1 | 0.19486895 | 0.335728479 |
| LIPT1 | PDHA1 | 0.098266486 | 0.096032823 |
| MTF1 | PDHA1 | 0.169086191 | 0.000436257 |
| GALNT14 | PDHB | -0.042276329 | 0.679365289 |
| CDKN2A | PDHB | 0.19611856 | 0.052940759 |
| DLAT | PDHB | 0.609713801 | 2.67719E-11 |
| DLD | PDHB | 0.537147193 | 1.18265E-08 |
| FDX1 | PDHB | 0.387563835 | 8.05648E-05 |
| GLS | PDHB | 0.416508872 | 1.99146E-05 |
| LIAS | PDHB | 0.218343757 | 0.030781018 |
| LIPT1 | PDHB | 0.240874982 | 0.016883374 |
| MTF1 | PDHB | 0.205847662 | 0.042004633 |
| PDHA1 | PDHB | 0.348545416 | 0.000436257 |
